# Proteins*Plus*: interactive analysis of protein–ligand binding interfaces

**DOI:** 10.1093/nar/gkaa235

**Published:** 2020-04-16

**Authors:** Katrin Schöning-Stierand, Konrad Diedrich, Rainer Fährrolfes, Florian Flachsenberg, Agnes Meyder, Eva Nittinger, Ruben Steinegger, Matthias Rarey

**Affiliations:** Universität Hamburg, ZBH - Center for Bioinformatics (ZBH), 20146 Hamburg, Germany; Universität Hamburg, ZBH - Center for Bioinformatics (ZBH), 20146 Hamburg, Germany; Universität Hamburg, ZBH - Center for Bioinformatics (ZBH), 20146 Hamburg, Germany; Universität Hamburg, ZBH - Center for Bioinformatics (ZBH), 20146 Hamburg, Germany; Universität Hamburg, ZBH - Center for Bioinformatics (ZBH), 20146 Hamburg, Germany; Universität Hamburg, ZBH - Center for Bioinformatics (ZBH), 20146 Hamburg, Germany; Universität Hamburg, ZBH - Center for Bioinformatics (ZBH), 20146 Hamburg, Germany; Universität Hamburg, ZBH - Center for Bioinformatics (ZBH), 20146 Hamburg, Germany

## Abstract

Due to the increasing amount of publicly available protein structures searching, enriching and investigating these data still poses a challenging task. The Proteins*Plus* web service (https://proteins.plus) offers a broad range of tools addressing these challenges. The web interface to the tool collection focusing on protein–ligand interactions has been geared towards easy and intuitive access to a large variety of functionality for life scientists. Since our last publication, the Proteins*Plus* web service has been extended by additional services as well as it has undergone substantial infrastructural improvements. A keyword search functionality was added on the start page of Proteins*Plus* enabling users to work on structures without knowing their PDB code. The tool collection has been augmented by three tools: StructureProfiler validates ligands and active sites using selection criteria of well-established protein–ligand benchmark data sets, WarPP places water molecules in the ligand binding sites of a protein, and METALizer calculates, predicts and scores coordination geometries of metal ions based on surrounding complex atoms. Additionally, all tools provided by Proteins*Plus* are available through a REST service enabling the automated integration in structure processing and modeling pipelines.

## INTRODUCTION

Available structural data of macromolecular complexes in the Protein Data Bank (PDB) (1) are often used as starting point for the successful development of new drugs (2). Although data quality and resolution increase with continuous improvement of methods, structure quality assessment, data enrichment and investigation are a prerequisite for successful structure-driven life science research. Selecting an appropriate macromolecular complex as starting structure poses a great challenge with regard to the growing number of available data and the great differences in quality and applied structure determination methods. Manually curated benchmark datasets like the Astex Diverse Set (3) or the Iridium HT (4) are outdated by now, but the selection criteria used for the generation of these sets are still applicable to the search for new reliable structures. In order to keep pace with the rate of data generation, there is a need for fully automated structure validation methods. The data selection step is followed by structure enrichment consisting of adding computed properties, which cannot be derived directly from the structure determination. A prominent example for an essential enrichment step is the addition of hydrogens to X-ray or Cryo-EM determined structures. The estimation of the formation of hydrogen bonds between protein and ligand directly depends on the calculated positions of hydrogens, protonation state, and the tautomeric state of the amino acid side chains and bound ligands. Also the correctness in prediction of water molecule positions plus the orientation of the water hydrogens and the assignment of metal coordination geometries are essential for a functional understanding of binding and influences the prospects of a design process.

Finding answers to the various questions emerging in a modeling process poses a great challenge for scientists. Many web services addressing specific topics like pocket detection (5), protein–ligand interaction visualization (6,7), protein–protein interface analysis (8,9) and metal interactions (10–12) exist. But there is a lack for comprehensive solutions offering different tools in a unified interface that facilitates the reuse of intermediate results and provides interoperability between tools. The members of the Worldwide PDB partnership (wwPDB; wwPDB.org) (13), for example, provide numerous tools and services to access and explore PDB content (14–16) on their own web pages. Additional to the web services, many software tools for protein structures and their complexes have been developed both as open source (17) and commercial solutions. Often, software usage is restricted by platform dependencies and installation obstacles. Web servers circumvent these issues, however, interoperability between tools and command line based applications remain problematic in practice.

**Figure 1.**
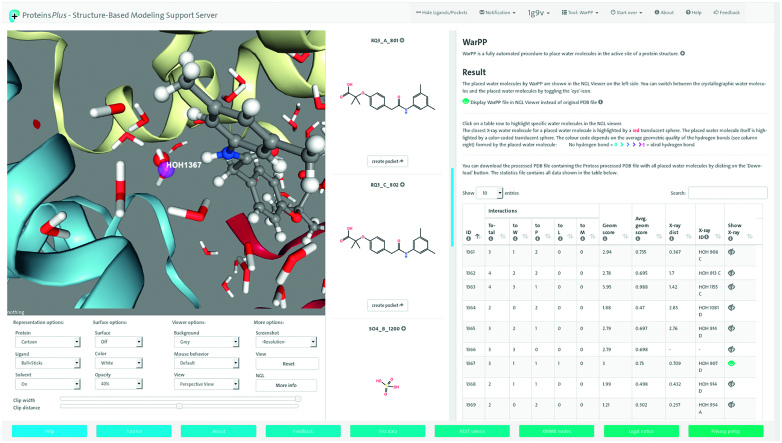
The Proteins*Plus* GUI. The 3D representation of human deoxy hemoglobin (Hb) complexed with RSR-13 (PDB code: 1G9V (41)) is shown on the left hand side together with the control panel for the NGL viewer options. The central panel contains a scrollable list of structure diagrams of all ligands contained in the PDB file. On the tool panel on the right hand side, the calculation results of WarPP are shown in a table. In the NGL viewer, a water molecule corresponding to one line in the table is shown. The red translucent sphere shows the position of the closest X-ray water molecule and the pink sphere denotes a good average hydrogen bond quality for this water molecule.

Here, we present an extended version of Proteins*Plus* that addresses a large variety of molecular modelling tasks covering the following areas: structure quality assessment by EDIA (18) and StructureProfiler (19), structure enrichment by Protoss (20), WarPP (21), METALizer, 2D visualization by PoseView (22), binding site ensemble generation by SIENA (23), protein–protein interface classification by HyPPI and pocket detection and druggability estimation by DoGSiteScorer (24). The web interface to our tool collection focusing on protein–ligand interactions has been geared towards easy and intuitive access for life scientists. This includes the visualization of the 3D structure in the embedded NGL viewer (25,26) and the 2D structure diagrams of all ions and small molecules. The layout of the start page with only a text field and two upload buttons is similar to the start page of popular internet search engines and therefore self-explanatory. Once the desired structure has been selected or uploaded, the default layout of the page consisting of the 3D view of the complex on the left hand side, a column containing the aforementioned structure diagrams in the middle of the page, and a tool panel on the right hand side is loaded, see Figure 1. The textual or tabular results of the different tools are presented on tool panel while the 3D view is updated accordingly in order to visualize the calculated result. Structure selections for the different calculations, e.g. a metal ion for running the METALizer (see below), can be done by clicking on the structures of interest in either the 2D or 3D representation. Clicking on results in the tool panel highlights or toggles the corresponding structure in the NGL viewer.

## MATERIALS AND METHODS—ENHANCEMENTS OF THE ProteinsPlus WEB SERVER

Since our last publication in 2017, the Proteins*Plus* web service has been extended by additional tools (WarPP, METALizer and StructureProfiler) as well as it has undergone substantial infrastructural improvements. Most noteworthy, a keyword search, interactive pocket definition and handling, and a REST API have been implemented. The keyword search enables the user to start Proteins*Plus* without knowing the PDB code of the structure of interest. StructureProfiler screens structures based on selection criteria typically used upon data set assembly for structure-based design methods. Combined with EDIA, a comprehensive structure validation is enabled within Proteins*Plus*. WarPP places water molecules for the active site of a given PDB file. METALizer predicts the metal coordination geometry and provides statistical information about the coordination distribution of metal ions in the PDB. The usage of the tools is visually supported within Proteins*Plus*. All tools can be used in an automated way via a REST service. The newly added functionality of Proteins*Plus* will be described in detail below.

### Data handling

#### Keyword search

The entry point to the Proteins*Plus* service is in many cases a publicly available structure from the PDB whose PDB code is not necessarily known to the user. To overcome this issue, a keyword search combined with a small number of quality filters was introduced. Searching with a keyword enables the user to find structures by e.g. a protein or author name, a ligand id or a SMILES string. The obtained results can be further filtered by the deposition date, the experimental method and resolution, and the organism. The keyword search is performed directly on the PDB via its RESTful Web Service APIs (https://www.rcsb.org/pdb/software/rest.do). The service provides different query types controlling the data fields considered. The initially used query type in Proteins*Plus* is ‘Text Search’ that searches all fields in each entry and can be refined afterwards by additional keyword searches in user-selected fields. All results are presented in a list sorted by the Match Score for the keyword. Interactive histograms give an overview on key data elements like resolution and deposition time and enable easy filtering. Besides summarizing textual information, a 3D picture of the whole structure and 2D structure diagrams of the ligands are provided. Next, with the selected structure the user can decide to start Proteins*Plus* with the default tool overview or directly with a specific tool.

#### Pocket handling

Most of the provided tools within the ProteinsPlus service perform their calculations on the binding site of the protein–ligand complex. Hence, a pocket definition functionality has been added. Pockets can be generated automatically from ligands, manually created by the user by selecting individual amino acids, or extracted from DoGSiteScorer (24) calculations. If a pocket is derived from a ligand, all amino acids in a radius of 6.5 Å to any ligand atom are selected following the recommendations concerning pocket sizes given in former publications (27,28). All pockets can be modified interactively by adding or removing amino acids. Several pocket definitions can be generated for the starting structure. Pockets can be visualized and used as input for binding site ensemble calculations with SIENA (23).

### Structure validation and selection

Up to date, high quality data sets for the validation of structure-based design methods are often manually curated. The growing amount of available structures and the need for specially tailored data sets requires an automated generation of such data sets. StructureProfiler (19) was developed as an all-in-one tool to screen structures based on selection criteria typically used in data set assembly for structure-based design methods. Combined with EDIA (18), which calculates an electron density score for individual atoms and was presented in our previous publication (29), a comprehensive structure validation is enabled within the Proteins*Plus* web service. The analysis performed by StructureProfiler can be divided into four different areas: First, the quality of the experimental data is evaluated using the resolution of the protein structure, its diffraction precision index, R and R_free factor, their difference, and the model significance. Secondly, the pocket around a ligand is analyzed for its occupancies, intramolecular clashes, EDIA_m_ per residue, deviations from standard VSEPR bond angles and usual bond lengths. Thirdly, the pocket to ligand B-factor ratio and their intermolecular clash is inspected. Last, small molecules in up to 8.0 Å distance to the protein complex are analyzed as ligands by StructureProfiler. With 21 tests ranging from EDIA_m_ over torsion angle analysis to their possible exclusion through a SMARTS and a ligand id (PDB HET code) filter, the features of the ligand can be well profiled. Overall, a thorough, objective, transparent, and automatic analysis of any complex available in the PDB can be performed with the help of StructureProfiler.

### Water molecules and metal ions

Water molecules and metal ions play a key role in the mediation of protein–ligand interactions. Therefore, the Proteins*Plus* tool suite has been augmented by a water placement procedure (WarPP) (21) and a metal complex geometry prediction tool (METALizer). WarPP, validated on ten thousands of crystallographic waters, places water molecules in the binding sites of a given PDB structure. METALizer predicts the metal coordination geometry and provides statistical information about metal coordination type distribution in the PDB.

WarPP predicts the energetically favorable, stable, positions of water molecules in protein–ligand binding sites. In a first step, free interaction directions are identified, which include nitrogen or oxygen atoms with an unsaturated hydrogen bond function. Additionally, hydrogen bond acceptors and donors with a bad geometry are considered (geometric score < 0.85, see (21)). Based on interaction geometries, previously derived from a large scale analysis of interactions in high resolution protein structures using NAOMInova (30,31), potential water positions are generated in ideal hydrogen bond distances (2.6 and 2.8 Å). These discrete points receive a geometric score based on their deviation angle to the ideal interaction direction. Next, the availability of these potential water positions needs to be determined. Due to close contact with other ligand or protein atoms, some of the interaction surface may not be available and thus cannot be converted into potential water positions. Finally, all potential water positions that are position-optimized and merged in a self-assembling procedure. Herein, based on the individual geometric score, the potential water positions are shifted towards each other until clusters are generated. These clusters are then used to predict water molecules whose location undergo a final numerical optimization.

In a second iteration, further water placement identifies water-water interactions in binding sites, which otherwise might not be identified and can contribute to important water networks. For more details on the WarPP method and its parametrization, please refer to our publication (21).

The web service displays the placed water molecules in the protein–ligand binding site. Additionally, important information regarding the formed hydrogen bonds to water molecules are summarized. If crystallographic water molecules were available in the starting structure, these water molecules will be used as a reference for the placed water molecules. The closest water molecule to each predicted one is available in a tabular representation and can also be displayed in the 3D view, see Figure 1.

**Figure 2.**
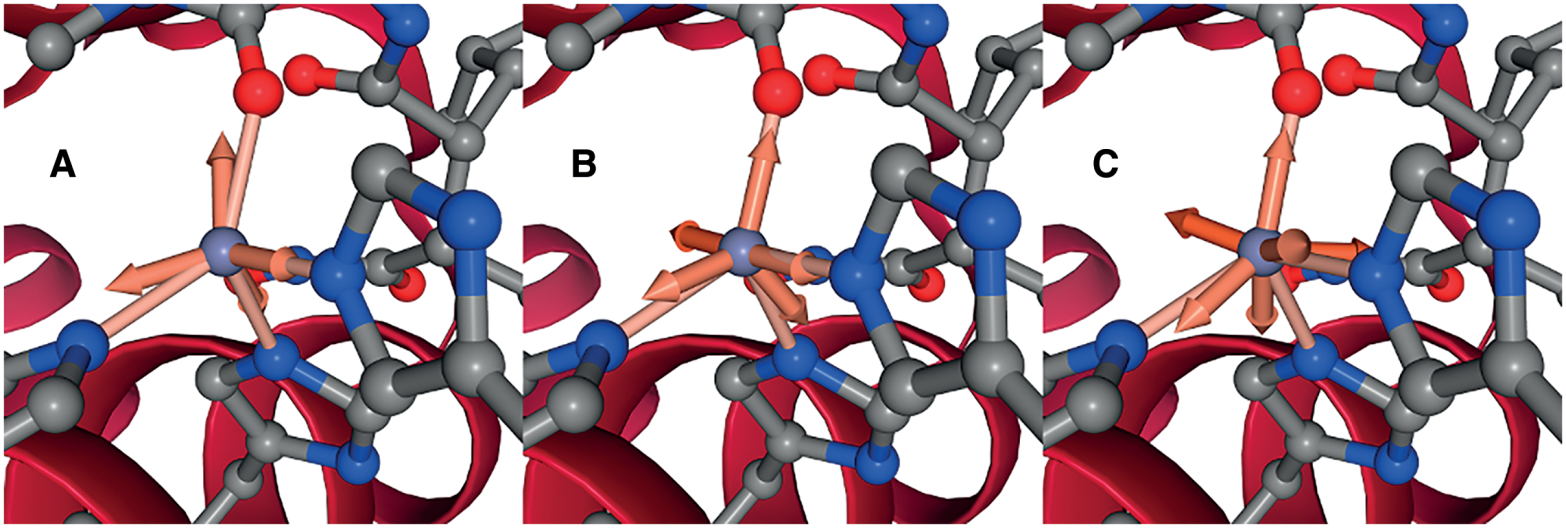
Visualization of METALizer results for Atrolysin C with Batimastat (PDB code: 1DTH (42)). The connection between the coordinating atoms and the metal ion are denoted by solid lines, the optimal geometry is denoted as arrows outgoing from the ion. METALizer predicts three different coordination geometries for the zinc ion bound to chain A of the protein: (**A**) tetrahedral (Free Sites: 0, Geometry RMSD: 0.190, Overlap Penalty: 0.0, Score: 9.51), (**B**) trigonal bipyramid (Free Sites: 1, Geometry RMSD: 0.173, Overlap Penalty: 0.0, Score: 12.63) and (**C**) trigonal prismatic (Free Sites: 2, Geometry RMSD: 0.246, Overlap Penalty: 0.001, Score: 20.29). The tetrahedral geometry is considered to be the best one due to the lowest calculated score.

METALizer is a tool to analyze the coordination geometry in protein–ligand complexes. In the Proteins*Plus* server METALizer is combined with EDIA (18) for additional quality assessments and SIENA (23) for the search for similar metal sites. Initially, METALizer identifies the coordinating atoms in the metal's coordination sphere; Supplementary Table S1 in the Supporting Information contains a list with element-specific radii of the coordination spheres. All oxygen, nitrogen, sulfur, and chlorine atoms are used as coordinating atoms; carboxylate groups are treated as potential bidentates (32). METALizer identifies the best fitting metal coordination geometries by superposing the geometric arrangement of the coordinating atoms in the binding site to ideal reference geometries (see Supporting Information, Supplementary Table S2 for a list). First, the RMSDs between the angle list of the query site and the angle lists of the reference geometries are calculated (32). For the selected reference geometries the actual superposition is calculated and the resulting distance RMSD is then used for scoring (33). The resulting coordination geometries are scored with a function that includes - besides the superposition RMSD—also the number of free coordination sites (preferring simple geometries) as well as the overlap that a potential binding partner at the free sites would have with other atoms in the protein–ligand complex (this parameter is also used, e.g. by UCSF Chimera (34)). The superposed coordination geometries are supplemented with statistics calculated on the PDB on the frequency of different coordination geometries for the given metal ion and the distribution of metal–partner distances. As an example, the superposition of a calculated zinc geometry and the three closest reference coordination geometries is shown in Figure 2.

Using EDIA (18) it can be checked how well each coordinating atom is supported by the electron density providing an additional quality assessment of the metal coordination site. SIENA (23) allows the fast retrieval, structural superposition and analysis of similar metal sites (with a sequence identity of ≥70% within the metal site) from the PDB. Within seconds to minutes similar metal sites can be retrieved using SIENA, analyzed and compared with METALizer, finding at least one similar site in another PDB structure for 75% of our test queries (for details, see Supporting Information). Additionally, the very same statistics as for the PDB (coordination geometry frequency and metal-partner distances) are calculated for the SIENA ensemble of similar metal sites.

METALizer provides the same basic functionalities (metal coordination geometry identification and statistics) as other—still maintained—web servers with a focus on metal ions in biological complexes like the MetalPDB (12) or the CheckMyMetal server (11) do, however, has some unique features making it complementary to existing tools: The integration of our EDIA score adds valuable information to the quality assessments given by the CheckMyMetal server. Our SIENA-based search for similar metal binding sites has a different focus than the MetalS^3^ (10) database search tool within the MetalPDB: The SIENA-based search together with METALizer is able to find and analyze metal sites with a similar amino acid sequence to the query metal site within seconds to minutes. On the other hand, the MetalS^3^ tool searches for metal sites that are structurally similar, however, can take hours to run for user-provided PDB files (10). For more information about computing times and search results of METALizer in combination with SIENA, see last paragraph and Supplementary Figure S1 in the Supporting Information.

### Accessibility

Additional to the graphical user interface, a REST API for each of the Proteins*Plus* tools has been made available. API requests can be sent with the command line tool curl or with a browser rest client plugin. The API allows the user to create jobs for the respective tools, each requiring a different set of parameters. Calculation results can then be accessed and downloaded. The base URL for version 1 is https://proteins.plus/api. The REST service usage and output is documented in detail for each individual tool on the Proteins*Plus* website together with a sample call for both the POST and the GET method. Providing a REST API makes the different tools available for an automated integration in modelling pipelines and software libraries. As an application example, a KNIME node (https://www.knime.com) has been developed for each tool and made available on the Proteins*Plus* website showcasing the usage of the respective APIs.

## SUMMARY AND OUTLOOK

Together with the additional functionality described above, Proteins*Plus* evolved into a versatile instrument for molecular modeling processes. The analysis and processing of binding sites and ligands on atomic level give comprehensive insights in the binding mode of the interacting molecules. In 2019, the server received 61,830 unique page view requests from 21,217 users. Further usability improvement of Proteins*Plus* workflows could be reached by an increase of tool interoperability: using results from calculations of other tools as input without needing intermediate formatting steps would enable the implementation of automated workflows.

Proteins*Plus* combines the advantages of a web service and a molecular modeling desktop application: the unified graphical user interface makes the usage of new or unfamiliar tools possible without a tedious learning effort, calculation results can be interconnected or reused for further calculations, and no local installation is needed. Connecting Proteins*Plus* to other web services could lead to deeper knowledge of a PDB structure. So far, a connection to the enzyme database BRENDA (35) already exists. A tool that searches for related bioactivity data of a complex in ChEMBL (36) is already included as alpha version in Proteins*Plus*. Currently, we investigate methods to include alternative structure files, for example from PDB-REDO (37).

For the near future, we plan to extend Proteins*Plus* by a search functionality that performs a textual, numerical and 3D search with full chemical awareness in protein–ligand interfaces (38). Additionally, we intend to incorporate docking and virtual screening methods (39,40). Thus, Proteins*Plus* opens the way to a large range of functionality from the analysis of protein structure and function to molecular design techniques for every life scientist.

## Supplementary Material

gkaa235_Supplemental_File

## Notes

Present address: Eva Nittinger, Medicinal Chemistry, Respiratory Inflammation and Autoimmune (RIA), BioPharmaceutical R&D, AstraZeneca, Gothenburg, Sweden; Ruben Steinegger, IT, Perfood GmbH, Lübeck 23564, Germany.
