# Supplementary material for: ProteinsPlus: interactive analysis of protein–ligand binding interfaces
Source: Nucleic Acids Res. 2020 Apr 16;48(W1):W48–53. doi: 10.1093/nar/gkaa235 (PMC7319454; doi:10.1093/nar/gkaa235)
Supplement: gkaa235_Supplemental_File [file gkaa235_supplemental_file.pdf]

Supporting Information for NAR Publication:

ProteinsPlus: Interactive Analysis of Protein-Ligand Binding Interfaces

Katrin Schöning-Stierand[1,*], Konrad Diedrich[1], Rainer Fährrolfes[1], Florian Flachsenberg[1], Agnes Meyder[1], Eva Nittinger[1], Ruben Steinegger[1] and Matthias Rarey[1,*]

Universität Hamburg, ZBH – Center for Bioinformatics, 22146 Hamburg, Germany

* To whom correspondence should be addressed. Tel: +49 40 42838-7350; Fax: +49 40 42838-7352; Email: stierand@zbh.uni-hamburg.de, rarey@zbh.uni-hamburg.de

METALizer Parameters: METALizer filters out any complex geometries with an angle list RMSD (1) of more than 21.5° and with more than 25 % free coordination sites. The cutoff distance for a coordinating atom is metal-specific and given in Table S1. The considered coordination geometries are given in Table S2.

Table S1. Default cutoff values for different metal atoms used in METALizer.

**Metal**	**Cutoff distance**
Mg	2.7 Å
Zn	2.8 Å
Fe	2.8 Å
Ca	3.4 Å
Na	3.5 Å
Mn	3.0 Å
K	3.5 Å
Cu	2.7 Å
Cd	3.4 Å
Sr	3.5 Å
Co	2.5 Å
Ni	2.9 Å
Hg	3.4 Å
Ba	3.5 Å
Pt	2.6 Å

Cs	3.5 Å
V	2.4 Å
Ru	2.4 Å
Other metals	3.0 Å

Table S2. Coordination geometries in METALizer with the coordination number of the fully coordinated geometry.

**Coordination geometry**	**Coordination number**
linear	2
trigonal planar	3
square planar	4
tetrahedral	4
square pyramid	5
trigonal bipyramid	5
octahedral	6
trigonal prismatic	6
pentagonal bipyramid	7
cubic	8
dodecahedral	8
square antiprismatic	8
none	-

SIENA Parameters: The SIENA search with metal site queries is performed with the "Flexibility analysis" presets in the Proteins*Plus* server (Site radius: 6.5 Å, Minimum fragment length: 10, Flexibility sensitivity: 0.6, Maximum fragment distance: 4, Minimum site identity: 0.7).

The search for and analysis of similar metal binding sites with SIENA and METALizer was evaluated on 6723 metal ions in the high resolution PDB subset published by Nittinger et al. (2) with at least one coordinating protein atom. Each metal ion was used as a query for SIENA against the PDB (as of 02/02/2020) and the time needed for the search for and METALizer analysis of similar metal sites was recorded. Experiments were performed on a compute cluster with machines equipped with Intel® Xeon® E5-4620 CPUs and 377 GB of RAM. The mean runtime is 40 s, the median runtime 13 s and

the runtime never exceeds 721 s, showing that the results can be obtained in seconds to minutes (and in most cases in less than one minute).

Furthermore, for each query the number of matching metal binding sites was determined (excluding potential matching bindings sites from the same PDB as the query). The results are shown in Figure S1. For more than 75 % of the queries at least one similar metal binding site can be found (excluding the sites from the query structure), in most cases even more results are obtained.

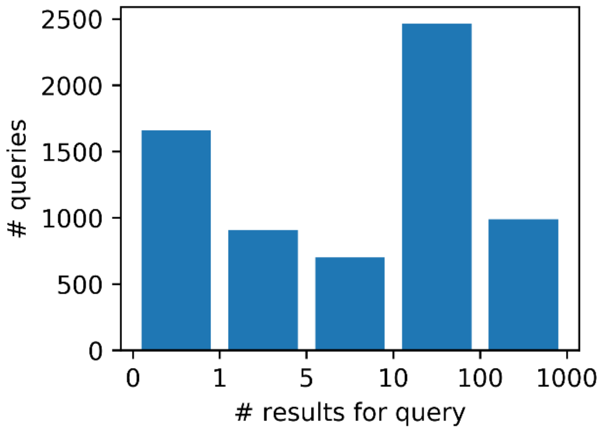

Figure S1. Histogram of the number of matching metal sites without matches within the query PDB structure.

REFERENCES

1. Seebeck,B., Reulecke,I., Kämper,A. and Rarey,M. (2008) Modeling of metal interaction geometries for protein-ligand docking. *Proteins Struct. Funct. Genet.*, 10.1002/prot.21818. https://doi.org/10.1002/prot.21818

2. Nittinger,E., Schneider,N., Lange,G. and Rarey,M. (2015) Evidence of water molecules - A statistical evaluation of water molecules based on electron density. *J. Chem. Inf. Model.*, **55**, 771–783. https://doi.org/10.1021/ci500662d
